# Reliability and Validity of the Linear Transducer “ADR Encoder” for Measuring Power and Speed of the Sit-to-Stand Tests in Older Adults

**DOI:** 10.3390/diagnostics15121475

**Published:** 2025-06-10

**Authors:** Luis Polo-Ferrero, María Luz Sánchez-Tocino, Arturo Dávila-Marcos, Marta Beatriz Carrera-Villegas, María Carmen Sánchez-Sánchez, Roberto Méndez-Sánchez

**Affiliations:** 1 Department of Nursing and Physiotherapy, University of Salamanca, 37007 Salamanca, Spain; pfluis@usal.es (L.P.-F.); mlsancheztocino@usal.es (M.L.S.-T.); turitodavila@usal.es (A.D.-M.); martacv28@usal.es (M.B.C.-V.); csanchez@usal.es (M.C.S.-S.); 2 Institute of Biomedical Research of Salamanca (IBSAL), 37007 Salamanca, Spain

**Keywords:** aged, frailty, geriatric assessment, muscle strength, reproducibility of results, validation study, physical fitness, cross-sectional studies

## Abstract

**Background/Objectives**: Lower-limb muscle function is traditionally assessed with tests like the five times sit-to-stand test (5STS) and the 30-s chair stand test (30CS). The ADR Encoder, a linear transducer, measures power and velocity during the sit-to-stand test. This study aimed to validate its use as functional indicators of muscle function in older adults. **Methods**: A cross-sectional study with 114 participants (mean age: 72.92 ± 4.97 years) was conducted. The 5STS, 30CS, power, and speed were measured using the ADR Encoder. Participants were categorized into frail, pre-frail, and robust groups according to the FRAIL Scale. Criterion, intraobserver, and interobserver validity were assessed. **Results**: Strong correlations (r > 0.7; *p* < 0.001) were found between power, speed, and both 5STS and 30CS. The interobserver intraclass correlation coefficient (ICC) for speed was 0.90 and the intraobserver ICC was 0.85. For power, the interobserver ICC was 0.88 and the intraobserver ICC was 0.79, all statistically significant (*p* < 0.001). Significant differences in power (7.78 W vs. 11.26 W; *p* < 0.001) and speed (0.40 m/s vs. 0.58 m/s; *p* < 0.001) were observed between frail and non-frail participants. **Conclusions**: The ADR Encoder is a valid, reliable tool for measuring power and speed in older adults. Its strong correlations with the 5STS and 30CS demonstrate its clinical relevance, with high reproducibility across observers and time points. Its application in diverse clinical and community settings could broaden its utility in geriatric functional assessment.

## 1. Introduction

Population aging, driven by rising life expectancy and the increasing proportion of older adults, presents significant challenges for healthcare systems worldwide. Among the most pressing concerns are the heightened prevalence of chronic conditions, progressive functional decline, and frailty [1]. Frailty is a clinical syndrome characterized by reduced physiological reserves and resilience, resulting in increased vulnerability to adverse outcomes such as falls, disability, hospitalization, institutionalization, and mortality [2]. It emerges from cumulative impairments across multiple organ systems and is often triggered by stressors that would be otherwise tolerated by healthier individuals [3]. Given its dynamic and potentially reversible nature—particularly in the initial stages—early identification and intervention are critical. In this context, assessing lower-limb muscle function plays a fundamental role in identifying individuals at risk and guiding the development of effective preventive and therapeutic strategies [4].

Lower-limb muscle function is commonly assessed through standardized functional tests such as the five times sit-to-stand test [5STS) and the 30-s chair stand test (30CS), both of which rely on the squat movement pattern—a key action in many daily living activities [5]. The 5STS quantifies the time required to complete five sit-to-stand repetitions, while the 30CS measures the maximum number of repetitions completed in 30 s [6,7]. Among the physical attributes assessed, muscle power—defined as the ability to produce force rapidly—is increasingly recognized as a critical determinant of functional performance and a more sensitive indicator of decline in older adults compared with strength alone [8,9].

Recent advances in technology have enabled the objective quantification of muscle power and velocity during functional tasks through the use of linear transducers. These devices, traditionally applied in sports science to evaluate athletic performance, are now being explored in geriatric populations as tools for detecting functional deficits and frailty. Linear transducers measure parameters such as peak velocity and power output during the concentric phase of movement, providing valuable insight into neuromuscular function. One such device, the ADR Encoder, has demonstrated validity in younger populations for measuring power and speed, supporting its potential utility in older adults as well [10].

Several studies have investigated the validity and reliability of linear transducers from various commercial brands for sit-to-stand testing (STS). Some have compared power and velocity outputs with gold-standard strength assessments, such as a unilateral leg extension on a leg press machine [11,12], while others have used alternative approaches like anchoring a belt around the participant’s waist to capture linear movement during the 5STS [13,14,15]. However, these methods present practical and methodological limitations. Leg press machines are not readily available in most clinical settings, and using waist-anchored belts can disrupt natural movement patterns, potentially compromising measurement accuracy due to non-linear trajectories and mechanical constraints.

Therefore, the present study aimed to validate the use of the ADR Encoder linear transducer for measuring power and velocity during the STS in older adults. Our goal was to determine whether this tool offers reliable and valid assessments of lower-limb muscle function, using a method that is accessible, reproducible, and applicable in clinical practice. Furthermore, we sought to compare its performance against established functional tests—namely the 5STS and 30CS—to examine its concurrent validity and potential for integration into routine geriatric assessment.

## 2. Materials and Methods

### 2.1. Study Design and Participants

A cross-sectional study was conducted in community-dwelling older adults to validate the measurement of speed and power during the sit-to-stand test (STS) using the ADR Encoder transducer (ADR Encoder, Toledo, Spain, version 5.2). These measures were compared with performance outcomes from the 5STS and 30CS to evaluate their consistency and clinical relevance. Participants were recruited from the local community of Salamanca as part of the supervised physical exercise program “PReGe.” Enrollment was voluntary, adhering to predefined eligibility criteria to ensure participant suitability. Initial assessments were conducted in January 2024, ensuring that participants had not engaged in supervised exercise sessions in the three months prior to the study to avoid potential residual effects of previous training.

Inclusion criteria required the participants to be aged 65 years or older, independently living in the community, capable of providing informed consent, and able to comprehend and complete the study assessments. Exclusion criteria included the presence of cardiac conditions, uncontrolled hypertension, recent surgery, acute pain for less than 15 days, or diagnosed neurological disorders.

The reporting of this study adheres to the CONSORT 2010 guidelines (Consolidated Standards of Reporting Trials) [16]. The trial protocol was approved on 13 June 2023 by the Ethical Committee for Research with Medicines of the Salamanca Health Area (code PI 2023061317) and was conducted in accordance with the principles of the Declaration of Helsinki [17]. The study was registered at ClinicalTrials.gov under the registration number NCT05870046.

To ensure representation across the full spectrum of frailty, participants were selected in equal numbers from the frail, pre-frail, and robust categories, according to the FRAIL Scale [18]. Randomization was performed using the Research Randomizer app (https://www.randomizer.org/) with a 1:1:1 ratio for frail, pre-frail, and robust participants.

### 2.2. Sample Size

The sample size was calculated on the basis of a previous study that validated the use of linear transducers for power assessment in the 5STS. The primary outcome was used with a 95% confidence interval (CI) and an intraclass correlation coefficient (ICC) range of 0.75–0.95, with a reported ICC of 0.93 [14,19]. On the basis of these parameters, an expected ICC of 0.90 and a lower bound of 0.80 were used. Considering an 80% probability of achieving the desired precision (0.2 total width) and a 10% dropout rate, the required sample size was estimated to be 58 participants. The calculation was performed using the GRANMO software, version 8.0.

### 2.3. Assessment and Outcomes

The assessment was conducted at the Research, Teaching, and Assistance Unit of the Faculty of Nursing and Physiotherapy at the University of Salamanca, a facility equipped to perform tests under optimal conditions. All tests were carried out by the same researcher following a standardized procedure. Prior to the tests, each participant was provided with an information sheet outlining the objectives, methodology, and expected outcomes of the study, with any questions answered verbally and voluntary consent obtained via a signature.

The evaluation began with the collection of demographic data, including the sex and age of each participant. Additionally, the participants were evaluated using the five-item FRAIL Scale, which includes questions on fatigue, resistance, ambulation, weight loss, and physical activity. On the basis of the number of positive responses, participants were categorized as frail (≥3), pre-frail (1–2), or robust (0) [18]. Regarding body composition variables, height was measured using a standard stadiometer, and weight and body mass index (BMI) were assessed using bioelectrical impedance analysis, Tanita BC-418 (TANITA Corp., Tokyo, Japan).

Functional assessments focused on lower-limb strength. A 5 min rest period was allowed between each test to prevent fatigue from affecting subsequent measurements. Power and velocity were measured using a linear transducer (ADR Encoder), and the results were compared with those from the 30CS and 5STS. All tests were performed on foldable chairs without armrests, with a height of 43.5 cm. In both the 30CS and 5STS, participants were instructed not to use their arms for assistance during the sit-to-stand movements and were required to cross their arms over their chest. Before starting the tests, the participants were allowed to perform 1 or 2 repetitions to familiarize themselves with the movement and ensure correct execution of the tests. This familiarization phase also served to verify the participants’ comprehension of the task and instructions, following established protocols.

#### 2.3.1. The 30-s Chair Stand Test (30CS)

The participants were instructed to perform the maximum number of complete sit-to-stand repetitions, emphasizing the importance of fully standing up and sitting down for the repetition to be considered valid. At the signal “go,” participants stood up completely, and the evaluator silently counted each elevation completed during the 30 s. The evaluator closely supervised the participant’s performance to ensure proper form. If the participant was fully standing or returning to a seated position, it was counted as one repetition. Incorrectly performed repetitions were not counted [7].

#### 2.3.2. Five Times Sit-to-Stand Test (5STS)

Participants were instructed to perform five complete sit-to-stand repetitions, returning to the seated position as quickly as possible. The timing began at the signal “go” and ended when the participant was fully seated after completing the fifth repetition [6].

#### 2.3.3. Power and Speed Measurement with the Linear Transducer

**Figure 1:**
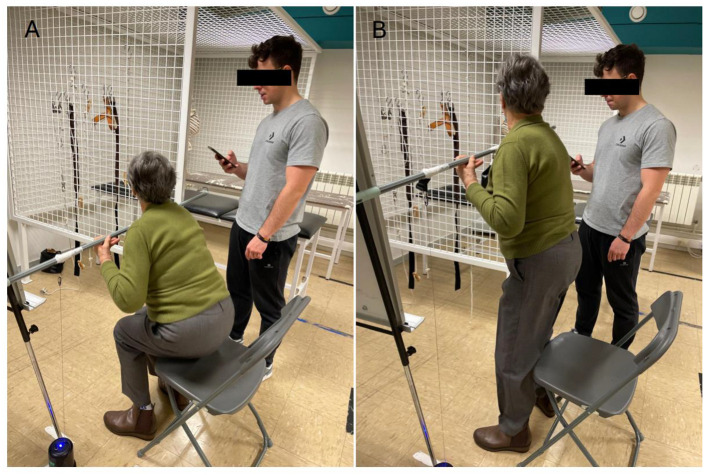
Performance of the test using the linear transducer. (**A**) Initial part of the concentric phase measurement during the linear transducer measurement. (**B**) Final phase of the concentric phase measurement during the linear transducer measurement.

The device was attached to a bar weighing 0.5 kg. Participants were instructed to hold the bar at shoulder height and to avoid any movement with the bar, simply guiding it with their hands while standing up and sitting back down, ensuring that the bar remained at shoulder height. The bar was kept in constant contact with two support pillars to ensure a completely linear movement (Figure 1). Participants were instructed to stand as quickly as possible for five repetitions, and the test began at the “go” signal and ended when the five rapid repetitions were completed. If any repetition showed a 25% difference from the other values, or if the bar was not in contact with the supports throughout the test, or if there was visible movement of the arms, the test was considered invalid and was repeated with rest allowed. The average values for speed (meters per second, m/s), power (watts, W), and distance (centimeters, cm) of the five repetitions were recorded using the linear transducer.

A second evaluator repeated the test using the linear transducer after 20 min to assess interobserver reliability. Between 5 and 10 days later, the initial evaluator repeated the test to assess intraobserver reliability, following the same methodology and instructions as the initial evaluation in both cases.

### 2.4. Statistical Analysis

An initial descriptive analysis was conducted to characterize the baseline variables of the participants. Continuous variables with a normal distribution were described as means ± standard deviations, and those without a normal distribution as medians (interquartile range, IQR). Categorical variables were expressed as frequencies and percentages. Data distribution was assessed using the Shapiro–Wilk test and box plots. Dependent variables (power, speed, 5STS, 30CS) with a normal distribution were analyzed using parametric tests; otherwise, a log-transformation was applied to approximate normality, and normality was re-verified.

To analyze the relationship of power and speed with performance in the 5STS and 30CS, Pearson correlations (r) were applied, classified according to Cohen’s guidelines (weak: 0.1–0.3; moderate: 0.4–0.6; strong: 0.7–1.0) [20]. Significant correlations were adjusted using partial analysis (r_p_), controlling for age and sex. Intra- and interobserver reliability were evaluated using the intraclass correlation coefficient (ICC, two-way mixed effects model) to estimate both consistency and agreement between repeated measurements, considering variability between observers and within the same observer. The level of agreement was interpreted according to Cohen’s criteria: poor reliability (<0.50), moderate (0.50–0.74), good (0.75–0.89), and excellent (>0.90) [21].

Linear regression analysis was used to estimate the unstandardized coefficient B, aiming to estimate the expected change in power or speed per unit of change in the 5STS or 30CS. Additionally, a comparison of variables between frail and non-frail individuals (pre-frail and robust groups) was performed using independent sample *t*-tests.

The significance level was set at *p* < 0.05 with a 95% confidence interval. Analyses were performed using IBM SPSS Statistics v28.

## 3. Results

### 3.1. Baseline Results

**Figure 2:**
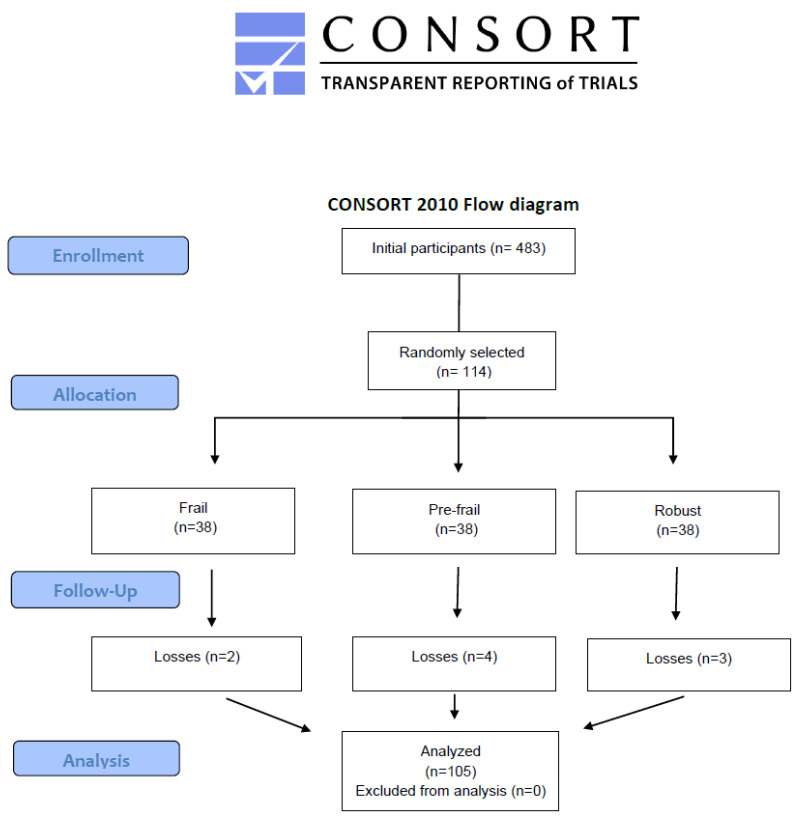
Flow diagram.

A total of 483 older adults were evaluated, and 114 participants were randomly selected in a 1:1:1 ratio of frail, pre-frail, and robust individuals (*n* = 38 per group). After excluding 9 participants due to missing data, the final sample comprised 105 participants (Figure 2).

The sample had a mean age of 72.9 ± 5.0 years and was composed of 56.6% females (*n* = 60). Descriptive statistics for all baseline variables are presented in Table 1. Normality was assessed using the Shapiro–Wilk test (Supplementary Material S1), revealing that BMI, speed, power, distance, and 5STS did not meet the normality assumption (*p* < 0.05). These variables were log-transformed to approximate normality, and re-evaluation confirmed normal distributions (*p* > 0.05). Consequently, parametric statistical methods were applied for further analyses.

### 3.2. Correlations of Speed and Power with Functional Performance in the 5STS and 30CS

The correlations of speed and power with the 5STS and 30CS were statistically significant (*p* < 0.001), as detailed in Table 2. All correlations were strong (*r* > 0.7), with speed demonstrating particularly notable associations. For the 5STS, speed exhibited a strong negative correlation with test times (*r* = −0.80, 95% CI: −0.86 to −0.71), which remained significant after adjusting for age and sex (*r_p_* = −0.71). In the 30CS, speed showed a strong positive correlation with performance (*r* = 0.80, 95% CI: 0.72 to 0.86), which also held after controlling for age and sex (*r_p_* = 0.72). These results indicate that both speed and power are critical determinants of functional performance in these tests.

### 3.3. Reliability of Speed and Power Measurements in 5STS and 30CS: Inter- and Intraobserver Consistency

The ICC demonstrated high inter- and intraobserver consistency for the measurements of speed and power concerning the 5STS and 30CS, with all values being statistically significant (*p* < 0.001), as detailed in Table 3. Notably, speed exhibited exceptional reliability: the interobserver ICC for speed was 0.90 (95% CI: 0.86–0.93), classified as excellent, while the intraobserver ICC was 0.85 (95% CI: 0.79–0.90), classified as good. In comparison, for power, the interobserver ICC was 0.88 (95% CI: 0.82–0.91), classified as good, and the intraobserver ICC was 0.79 (95% CI: 0.71–0.85), also classified as good, with all results being statistically significant (*p* < 0.001). These findings underscore the exceptional reliability of speed measurements and demonstrate that power, although slightly lower in reliability, also remains a consistent and reliable measure for assessing functional performance in older adults.

### 3.4. Regression Coefficients of Speed and Power

The regression coefficients (B) indicate the change in 5STS or 30CS scores for each unit of change in speed or power, as detailed in Table 4. For 5STS, speed had a negative effect (B = −0.036, 95% CI: −0.042 to −0.030), meaning each 1-second increase in time resulted in a 0.036 m/s decrease in speed, with statistical significance (*p* < 0.001). Power also had a negative effect (B = −0.71, 95% CI: −0.83 to −0.58), where each 1 s increase in 5STS time was associated with a 0.71-watt decrease in power (*p* < 0.001). In the 30CS, speed had a positive effect (B = 0.024, 95% CI: 0.020 to 0.027), with each one-repetition increase in 30CS corresponding to a 0.024 m/s increase in STS speed (*p* < 0.001). Power also showed a similar positive effect (B = 0.46, 95% CI: 0.39 to 0.54), where each one-repetition increase in 30CS resulted in a 0.46-watt increase in power, also statistically significant (*p* < 0.001). All relationships were statistically significant (*p* < 0.001).

### 3.5. Comparative Analysis of Frail and Non-Frail Older Adults

Table 5 presents the characteristics of frail and non-frail participants, including their statistical significance. Significant differences were observed in the 5STS and 30CS tests (*p* < 0.001), both recognized as predictive measures of frailty. Additionally, significant differences were found in power (7.78 ± 0.73 W vs. 11.26 ± 1.76 W; *p* < 0.001) and speed (0.40 ± 0.04 m/s vs. 0.58 ± 0.08 m/s; *p* < 0.001), indicating their potential as indicators of frailty. These findings emphasize the utility of functional tests, such as the 5STS and 30CS, along with power and speed assessments, in identifying frailty among older adults.

## 4. Discussion

The results of this study confirm that the linear transducer ADR Encoder is a valid device for measuring power and speed in older adults, showing significant correlations with widely used functional tests such as the 5STS and 30CS. These findings are consistent with previous research that has evaluated similar devices, such as the GymAware™ and MuscleLab, solidifying the role of linear transducers in the assessment of functional performance [13,14,15]. The strong correlations observed for power and speed with functional testing reinforce the importance of these variables as robust indicators of functional performance in older adults. Specifically, higher levels of power and speed were consistently associated with better performance on the 5STS and 30CS, supporting their utility in assessing physical functionality in this population [22]. These results are particularly relevant in the context of frailty and other aging-related conditions, where loss of muscle power has a significant impact on daily functionality [8,23].

Furthermore, this study supports the reliability and reproducibility of power and velocity measurements obtained with the ADR Encoder during STS, both in intraobserver and interobserver assessments, as demonstrated in the 5STS and 30CS [7,24]. The ADR Encoder showed comparable results to other linear transducers, such as the GymAware™ and MuscleLab. For example, previous research with the GymAware™ reported high correlations in power measurement during STS and 5STS, findings that are congruent with those presented in this study [14,15]. Likewise, MuscleLab has been shown to be effective in power and velocity measurement, which reinforces the validity of linear transducers in general for these applications [13]. Previous studies have established significant correlations between power and 5STS in populations spanning a wide age range, from young adults to the elderly, supporting the validity of these devices in diverse population groups [25].

Our study extends existing knowledge by validating the application of the ADR Encoder in a homogeneous and larger sample of older adults, which reinforces the robustness of the results obtained. These results highlight the practical value of the ADR Encoder as a feasible instrument for routine clinical assessments of lower-limb muscle function in older adults. Unlike previous studies, where the measurement of velocity and power with linear transducers was performed by anchoring the device to the waist, our methodology introduces a more correct perspective by addressing the limitations of this technique. When analyzing the motion performed by the encoder during the STS, it was observed that it is not purely linear. Therefore, our proposed methodology minimizes possible biases in the measurement and offers a more accurate alternative by allowing the encoder to perform the purely linear guided motion. This improvement in technique not only benefits the validation of the ADR Encoder but also opens the door to future research evaluating other linear transducers under these conditions. As reported in previous studies using leg press machines to assess muscle power in older adults [11,12], our findings suggest that similarly valid measurements can be obtained using simpler, more accessible equipment such as the ADR Encoder. We demonstrated that power assessments can be conducted accurately, affordably, and with minimal equipment, making them feasible in both clinical and research settings. The ADR Encoder, with a significantly lower cost (USD 250) compared with other validated devices, offers a practical and accessible alternative for healthcare professionals and researchers. The high reproducibility values, classified as good or excellent, further support the clinical utility of the ADR Encoder, ensuring consistent results regardless of the observer or assessment time. This reliability makes it a strong candidate for becoming a standardized method to assess speed and power during the STS, enabling the creation of normative reference data. Such a standard would provide healthcare professionals with an objective tool to interpret results and inform clinical decision-making.

The random inclusion of frail, pre-fragile and robust older adults, according to the FRAIL Scale, brings significant strength to the present study, as it allows generalization of the results to the entire older population. Similar to other physical performance tests that relate loss of physical function to frailty [26,27,28,29], our findings highlight significant differences in STS speed and power between frail and non-frail adults. These differences reinforce the potential of these parameters as sensitive indicators of muscle function loss and frailty. In future research, it would be valuable to establish specific cut-off points for speed and power to predict frailty in older adults, extending their utility beyond the sporting arena into broader clinical applications.

It is important to acknowledge the limitations of the present work. The initial assessment did not include a comprehensive evaluation of specific comorbidities or diseases, being limited only to the use of the FRAIL Scale. In addition, other functional tests, beyond the 5STS and 30CS, were not incorporated, as previous studies that have explored the correlation of linear transducers with various measures of physical performance have carried out [14,15,25,30]. Therefore, future research should compare the speed and power results obtained with the ADR Encoder with other standardized functional tests and explore its applicability in different clinical and community settings, which would allow for a more comprehensive assessment of its versatility.

The ADR Encoder is an effective tool for functional assessment and offers the potential for continuous, accurate monitoring of physical fitness in geriatric patients. This is crucial for personalized rehabilitation and early detection of mobility impairments, enabling timely interventions [11,12]. Its intuitive design ensures ease of use by healthcare professionals without extensive training. The device’s portability and affordability make it ideal for clinical and community settings, particularly in resource-limited areas. It can also be used in home visits, expanding its reach to patients with limited mobility or those unable to travel.

From a clinical standpoint, our experience using the ADR Encoder in daily practice has been highly positive. The device is easy to integrate into existing functional assessment routines, requiring minimal training for health professionals and no discomfort for older adults. In our setting, we have used it not only in research but also in supervised physical exercise programs, allowing us to detect subtle changes in power and speed over time that might not be captured by conventional tests. These real-time, objective measurements have enhanced our capacity to personalize interventions and track progress in a more sensitive and motivating way for participants. Furthermore, the affordability and portability of the device make it a feasible solution for broader clinical implementation, particularly in resource-constrained settings such as primary care or home-based programs.

## 5. Conclusions

The ADR Encoder has proven to be a valid and reliable device for measuring power and speed as functional indicators in older adults. The results show strong correlations with widely used functional tests, such as the 5STS and 30CS, reinforcing its clinical relevance. In addition, the device has demonstrated high reproducibility, both in assessments by different observers and at different times. Future research should focus on establishing cut-off points for speed and power to identify frailty in older adults. It would also be valuable to explore its application in a variety of clinical and community settings, expanding the scope and utility of this device in geriatric functional assessments.

## Figures and Tables

**Table 1:** Baseline characteristics of the participants.

Variable	Mean ± SD
Female, % ^a^	60 (56.6%)
Age, years	72.9 ± 5.0
Weight, kg	68.2 ± 10.4
Height, m	1.57 ± 0.09
BMI, kg/m^2 b^	27.4 (24.4, 31.3)
Speed, m/s ^b^	0.52 (0.36, 0.62)
Power, w ^b^	10.0 (7.0, 13.0)
Distance, cm	49.6 ± 8.6
5STS, s ^b^	10.5 (7.4, 13.5)
30CS	14.0 ± 4.0

^a^ Values are expressed as frequencies and percentages (%); ^b^ values are expressed as the median and interquartile range (IQR). SD, standard deviation.

**Table 2:** Spearman’s correlation coefficients and partial correlations for speed and power.

	5STS			30CS		
	*r*	*r_p_*	*p*	*r*	*r_p_*	*p*
Speed	−0.80 (−0.86–−0.71)	−0.71	<0.001	0.80 (0.72–0.86)	0.72	<0.001
Power	−0.76 (−0.84–−0.69)	−0.69	<0.001	0.78 (0.70–0.85)	0.70	<0.001

*r*, Spearman’s correlation coefficient; *r_p_*, partial correlation coefficient adjusted for age and sex; *p*, *p*-value for *r* and *r_p_*.

**Table 3:** Inter- and intraobserver reliability for speed and power measurement.

Variables	Interobserver ICC (95% CI)	*p*	Intraobserver ICC (95% CI)	*p*
Speed	0.90 (0.86–0.93)	<0.001	0.85 (0.79–0.90)	<0.001
Power	0.88 (0.82–0.91)	<0.001	0.79 (0.71–0.85)	<0.001

**Table 4:** Regression coefficients for speed and power in the 5STS and 30CS.

	B 5STS (95% CI)	*p*	B 30CS (95% CI)	*p*
Speed	−0.036 (−0.042–−0.03)	<0.001	0.024 (0.020–0.027)	<0.001
Power	−0.71 (−0.83–−0.58)	<0.001	0.46 (0.39–0.54)	<0.001

B, unstandardized regression coefficient.

**Table 5:** Functional performance and physical measures in frail vs. non-frail adults.

Variables	Frail (*n* = 36)	Non-Frail (*n* = 69)	*p*
Speed, m/s	0.40 ± 0.04	0.58 ± 0.08	<0.001
Power, w	7.8 ± 0.7	11.3 ± 1.8	<0.001
Distance, cm	48.2 ± 7.8	47.9 ± 7.9	0.567
5STS, s	14.7 ± 2.0	9.7 ± 1.3	<0.001
30CS	9.8 ± 2.0	15.9 ± 2.6	<0.001

Values are expressed as the mean and standard deviation; *p*: *t* tests for independent samples.

## Data Availability

The datasets generated and/or analyzed during the current study are available from the corresponding author on reasonable request. Data are not publicly available due to privacy and ethical restrictions.

## Supplementary Materials

The following supporting information can be downloaded at https://www.mdpi.com/article/10.3390/diagnostics15121475/s1. Supplementary Material S1. Normality tests and box plots.

## Abbreviations

The following abbreviations are used in this manuscript.

5STS	Five Times Sit-to-Stand Test
30CS	30-s Chair Stand Test
BMI	Body Mass Index
CI	Confidence Interval
ICC	Intraclass Correlation Coefficient
IQR	Interquartile Range
STS	Sit-to-Stand Test

## References

[1] Mitchell E., Walker R. (2020). Global ageing: Successes, challenges and opportunities. Br. J. Hosp. Med..

[2] Hoogendijk E.O., Afilalo J., Ensrud K.E., Kowal P., Onder G., Fried L.P. (2019). Frailty: Implications for clinical practice and public health. Lancet.

[3] Fried L.P., Tangen C.M., Walston J., Newman A.B., Hirsch C., Gottdiener J., Seeman T., Tracy R., Kop W.J., Burke G. (2001). Frailty in older adults: Evidence for a phenotype. J. Gerontol. A Biol. Sci. Med. Sci..

[4] Acosta-Benito M.Á., Martín-Lesende I. (2022). Fragilidad en atención primaria: Diagnóstico y manejo multidisciplinar. Aten. Primaria.

[5] Uysal I., Cetisli-Korkmaz N., Cavlak U. (2020). Assessment of the musculoskeletal performance with squat tests and performance-oriented measurements in older adults. J. Back Musculoskelet. Rehabil..

[6] Goldberg A., Chavis M., Watkins J., Wilson T. (2012). The five-times-sit-to-stand test: Validity, reliability and detectable change in older females. Aging Clin. Exp. Res..

[7] Jones C.J., Rikli R.E., Beam W.C. (1999). A 30-s chair-stand test as a measure of lower body strength in community-residing older adults. Res. Q. Exerc. Sport.

[8] El Hadouchi M., Kiers H., De Vries R., Veenhof C., Van Dieën J. (2022). Effectiveness of power training compared to strength training in older adults: A systematic review and meta-analysis. Eur. Rev. Aging Phys. Act..

[9] Skelton D.A., Greig C.A., Davies J.M., Young A. (1994). Strength, power and related functional ability of healthy people aged 65-89 years. Age Ageing.

[10] Lopez-Torres O., Fernandez-Elias V.E., Li J., Gomez-Ruano M.A., Guadalupe-Grau A. Validity and Reliability of A New Low-Cost Linear Position Transducer to Measure Mean Propulsive Velocity: The ADR device. Proc. Inst. Mech. Eng. P J. Sports Eng. Technol..

[11] Alcazar J., Losa-Reyna J., Rodriguez-Lopez C., Alfaro-Acha A., Rodriguez-Mañas L., Ara I., García-García F.J., Alegre L.M. (2018). The sit-to-stand muscle power test: An easy, inexpensive and portable procedure to assess muscle power in older people. Exp. Gerontol..

[12] Alcazar J., Kamper R.S., Aagaard P., Haddock B., Prescott E., Ara I., Suetta C. (2020). Relation between leg extension power and 30-s sit-to-stand muscle power in older adults: Validation and translation to functional performance. Sci. Rep..

[13] Lindemann U., Farahmand P., Klenk J., Blatzonis K., Becker C. (2015). Validity of linear encoder measurement of sit-to-stand performance power in older people. Physiotherapy.

[14] Balachandran A.T., Orange S.T., Wang Y., Lustin R., Vega A., Quiles N. (2024). Comparison of two popular transducers to measure sit-to-stand power in older adults. PLoS ONE.

[15] Orange S.T., Metcalfe J.W., Liefeith A., Jordan A.R. (2020). Validity of various portable devices to measure sit-to-stand velocity and power in older adults. Gait Posture.

[16] Moher D., Hopewell S., Schulz K.F., Montori V., Gøtzsche P.C., Devereaux P.J., Elbourne D., Egger M., Altman D.G. (2012). CONSORT 2010 explanation and elaboration: Updated guidelines for reporting parallel group randomised trials. Int. J. Surg..

[17] Association W.M. (2013). World Medical Association Declaration of Helsinki: Ethical Principles for Medical Research Involving Human Subjects. JAMA.

[18] Fried L.P., Ferrucci L., Darer J., Williamson J.D., Anderson G. (2004). Untangling the concepts of disability, frailty, and comorbidity: Implications for improved targeting and care. J. Gerontol. A Biol. Sci. Med. Sci..

[19] Borg D.N., Bach A.J.E., O’Brien J.L., Sainani K.L. (2022). Calculating sample size for reliability studies. PM&R.

[20] Cohen J. (2013). Statistical Power Analysis for the Behavioral Sciences.

[21] Cohen J. (1960). A Coefficient of Agreement for Nominal Scales. Educ. Psychol. Meas..

[22] Reid K.F., Fielding R.A. (2012). Skeletal muscle power: A critical determinant of physical functioning in older adults. Exerc. Sport Sci. Rev..

[23] Bean J.F., Leveille S.G., Kiely D.K., Bandinelli S., Guralnik J.M., Ferrucci L. (2003). A comparison of leg power and leg strength within the InCHIANTI study: Which influences mobility more?. J. Gerontol. A Biol. Sci. Med. Sci..

[24] De Melo T.A., Duarte A.C.M., Bezerra T.S., França F., Soares N.S., Brito D. (2019). The Five Times Sit-to-Stand Test: Safety and reliability with older intensive care unit patients at discharge. Rev. Bras. Ter. Intensiva.

[25] Ruiz-Cárdenas J.D., Rodríguez-Juan J.J., Smart R.R., Jakobi J.M., Jones G.R. (2018). Validity and reliability of an iPhone App to assess time, velocity and leg power during a sit-to-stand functional performance test. Gait Posture.

[26] Bischoff H.A., Stähelin H.B., Monsch A.U., Iversen M.D., Weyh A., von Dechend M., Akos R., Conzelmann M., Dick W., Theiler R. (2003). Identifying a cut-off point for normal mobility: A comparison of the timed “up and go” test in community-dwelling and institutionalised elderly women. Age Ageing.

[27] Pavasini R., Guralnik J., Brown J.C., Di Bari M., Cesari M., Landi F., Vaes B., Legrand D., Verghese J., Wang C. (2016). Short Physical Performance Battery and all-cause mortality: Systematic review and meta-analysis. BMC Med..

[28] Studenski S., Perera S., Patel K., Rosano C., Faulkner K., Inzitari M., Brach J., Chandler J., Cawthon P., Connor E.B. (2011). Gait speed and survival in older adults. JAMA.

[29] Cruz-Jentoft A.J., Bahat G., Bauer J., Boirie Y., Bruyère O., Cederholm T., Cooper C., Landi F., Rolland Y., Sayer A.A. (2019). Sarcopenia: Revised European consensus on definition and diagnosis. Age Ageing.

[30] Sherwood J.J., Inouye C., Webb S.L., Jenny O. (2019). Reliability and Validity of the Sit-to-Stand as a Muscular Power Measure in Older Adults. J. Aging Phys. Act..

