# Supplementary material for: Reliability and Validity of the Linear Transducer “ADR Encoder” for Measuring Power and Speed of the Sit-to-Stand Tests in Older Adults
Source: Diagnostics (Basel). 2025 Jun 10;15(12):1475. doi: 10.3390/diagnostics15121475 (PMC12199191; doi:10.3390/diagnostics15121475)
Supplement: Supplementary file 1 [file diagnostics-15-01475-s001.zip › diagnostics-3604412-supplementary.pdf]

Normality Tests.

	Kolmogorov-Smirnov[a]			Shapiro-Wilk		
	Est.	gl	Sig.	Est.	gl	Sig.
SEX	,374	106	<,001	,630	106	<,001
AGE	,073	106	,200*	,979	106	,088
WEIGHT	,054	106	,200*	,987	106	,396
HEIGHT	,100	106	,012	,984	106	,229
Body Mass Index	,069	106	,200*	,957	106	,002
Frailty	,231	106	<,001	,792	106	<,001
Speed	,077	106	,131	,958	106	,002
Power	,109	106	,004	,953	106	<,001
Distance	,064	106	,200*	,976	106	,047
Five sit to stand Test	,094	106	,023	,957	106	,002
Chair Stand Test	,070	106	,200*	,982	106	,159

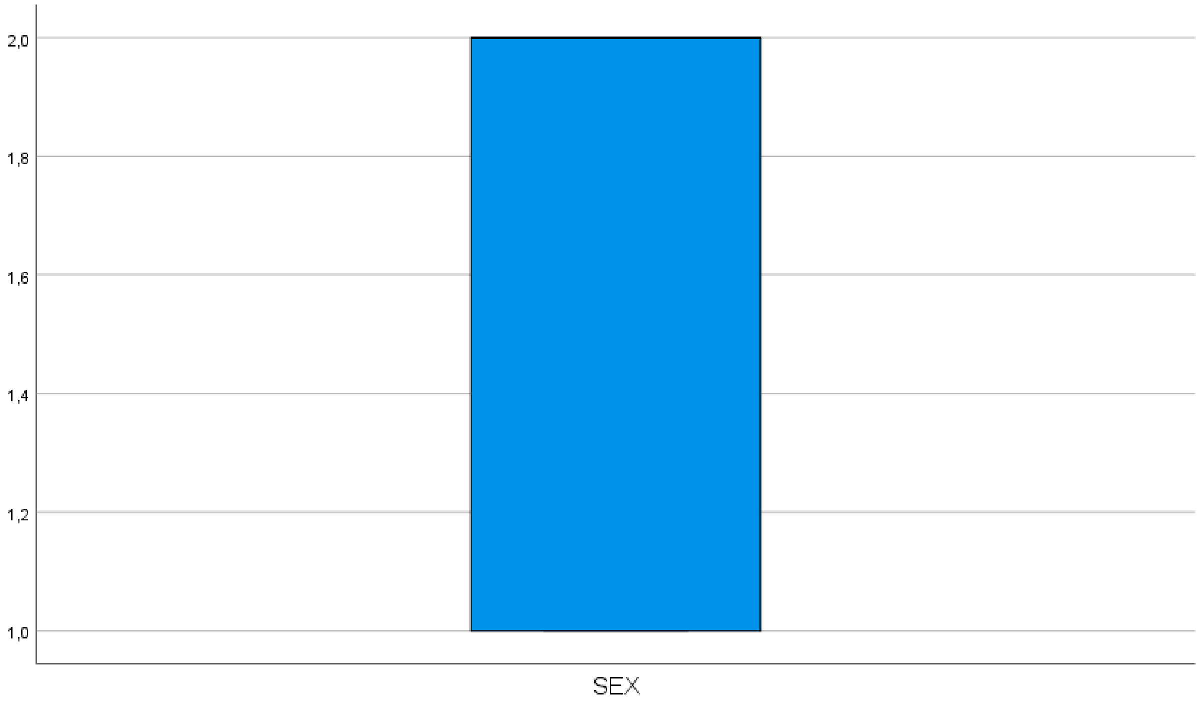
2,0
1,8
1,6
1,4
1,2
1,0
SEX

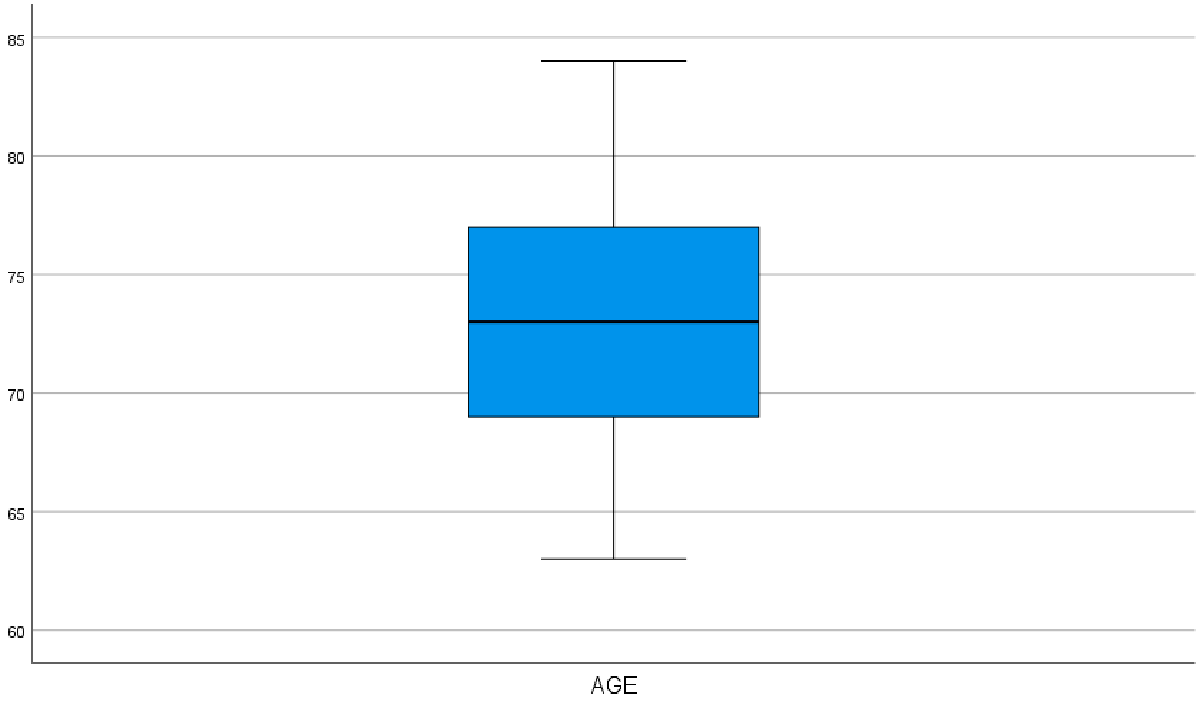
85
80
75
70
65
60
AGE

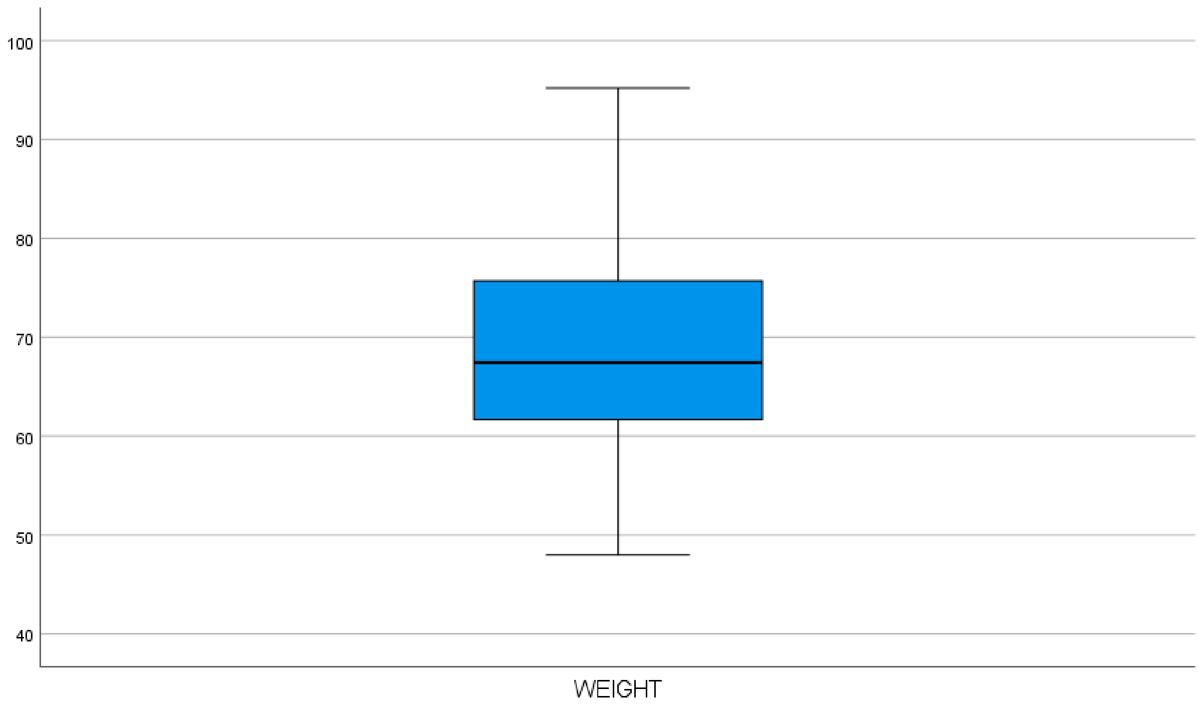
100
90
80
70
60
50
40
WEIGHT

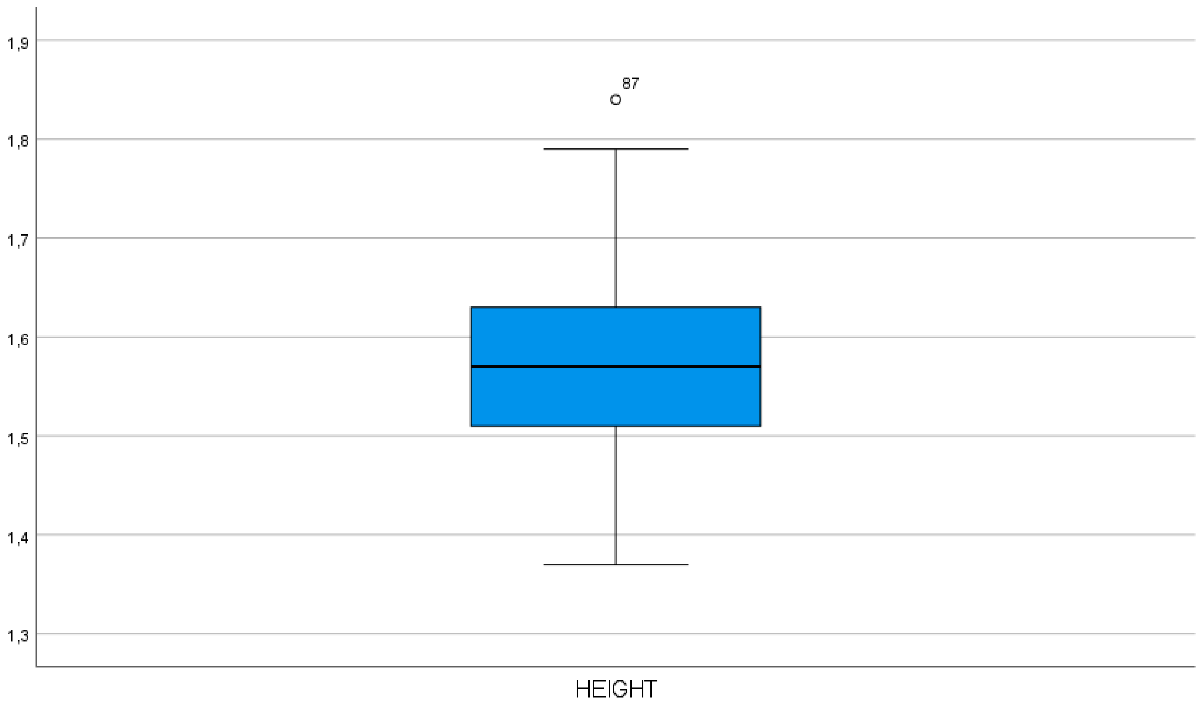
1,9
1,8
1,7
1,6
1,5
1,4
1,3
87
HEIGHT

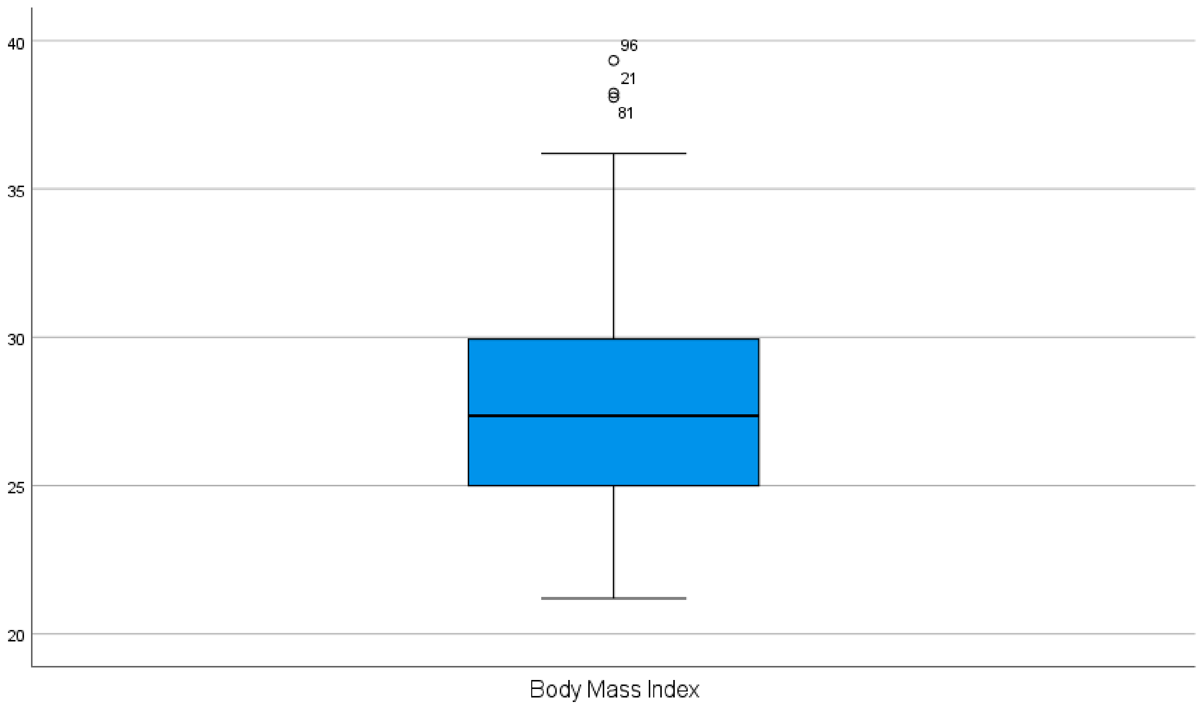
40
35
30
25
20
96
21
81
Body Mass Index

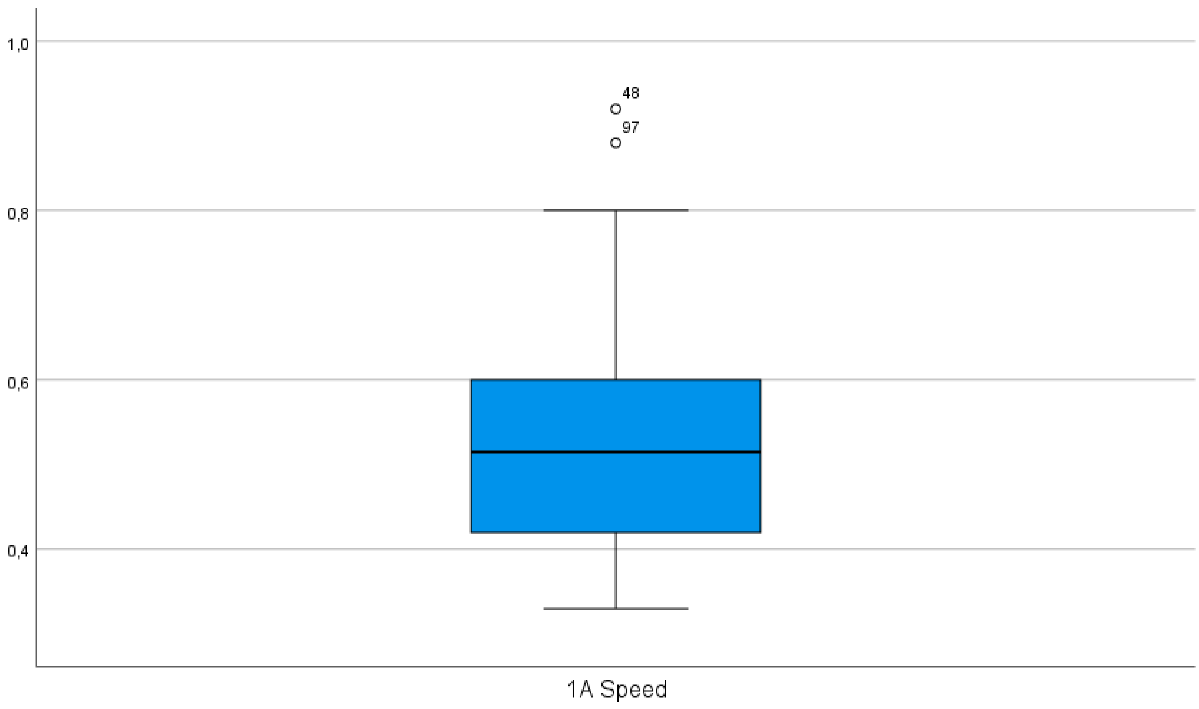
1,0
0,8
0,6
0,4
48
97
1A Speed

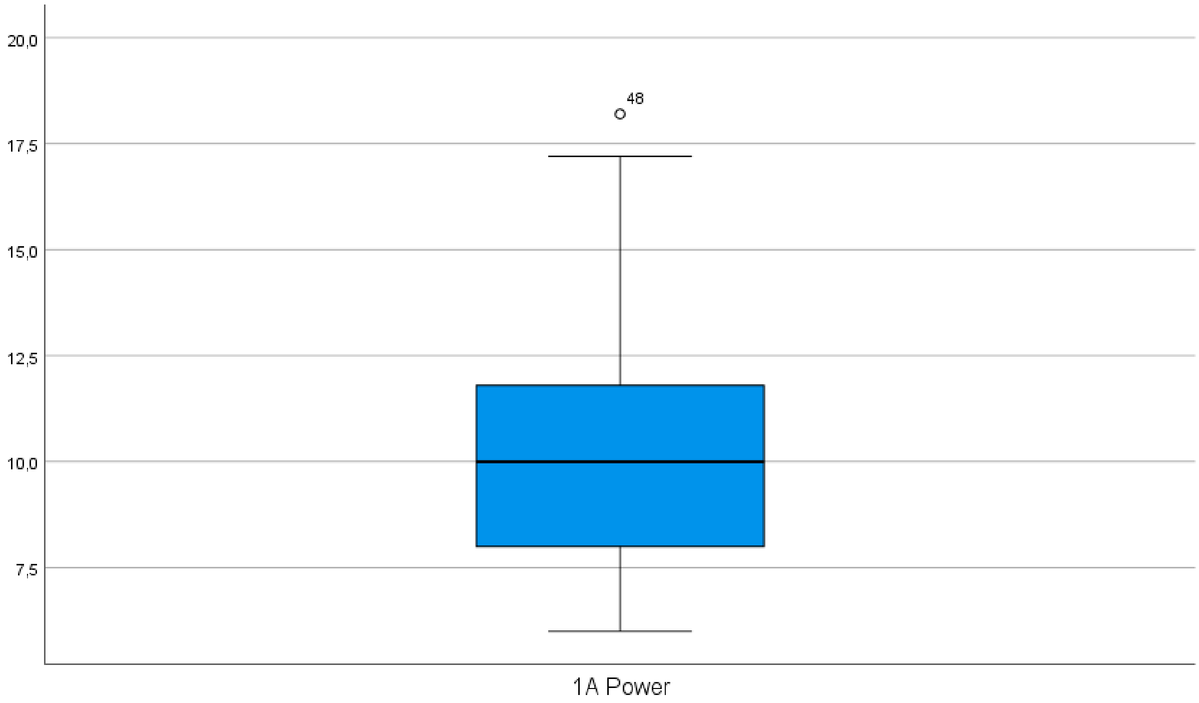
20,0
17,5
15,0
12,5
10,0
7,5
48
1A Power

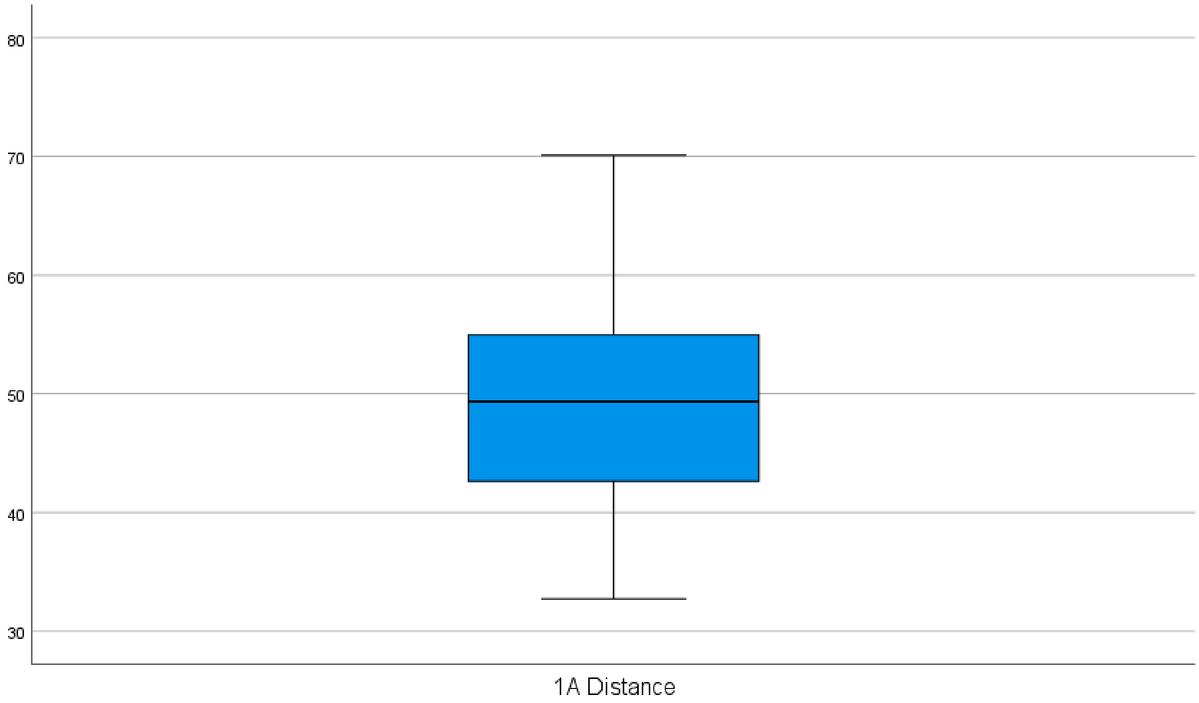
80
70
60
50
40
30
1A Distance

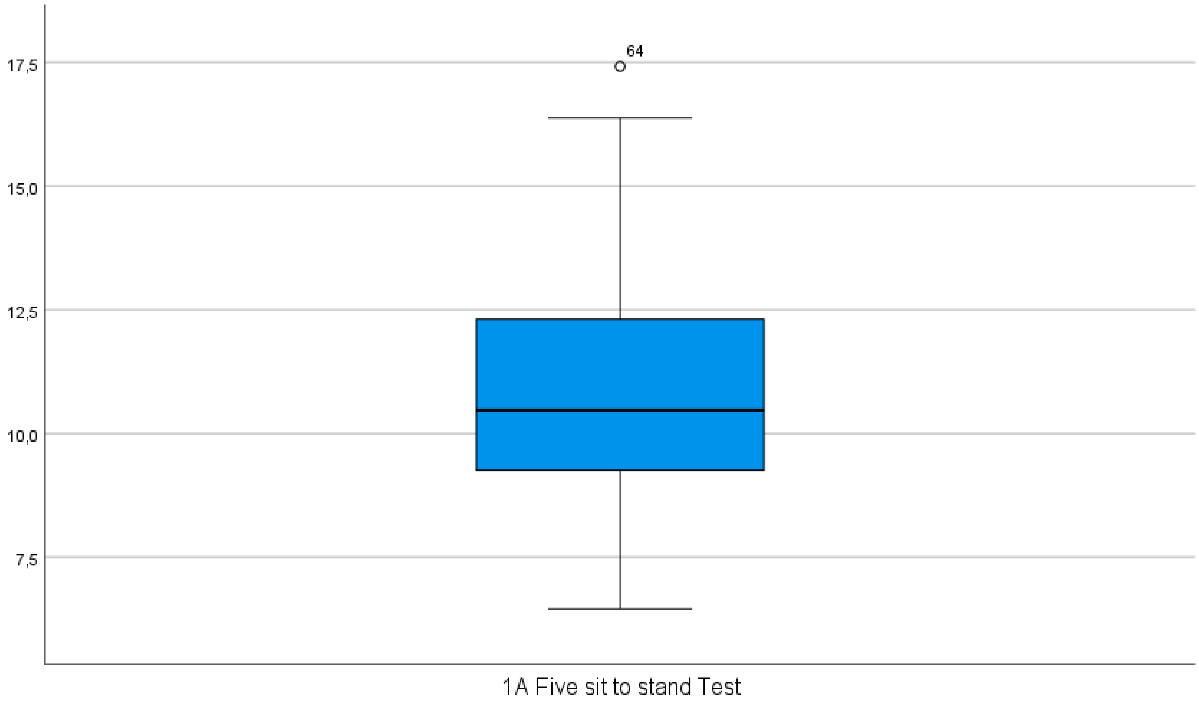
17,5
15,0
12,5
10,0
7,5
64
1A Five sit to stand Test

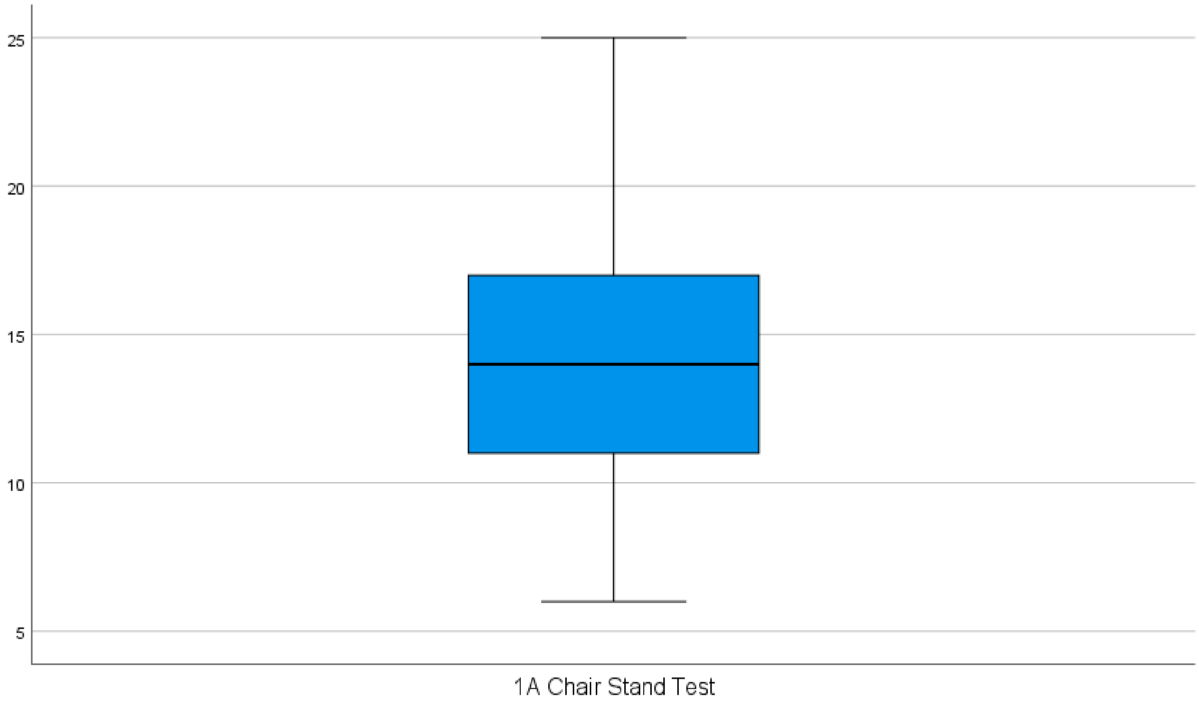
25
20
15
10
5
1A Chair Stand Test
